# Innovation as a value in healthcare priority-setting: the UK experience

**DOI:** 10.1007/s11211-019-00333-9

**Published:** 2019-04-15

**Authors:** Victoria Charlton, Annette Rid

**Affiliations:** 1 Department of Global Health & Social Medicine, King’s College London, 40 Aldwych, London, WC2B 4BG UK; 2 Department of Bioethics, The Clinical Center, U.S. National Institutes of Health, Bethesda, USA

**Keywords:** Healthcare priority-setting, National Institute for Health and Care Excellence (NICE), Social values, Innovation, Justice, Health equity, Accountability

## Abstract

All healthcare systems operate with limited resources and therefore need to set priorities for allocating resources across a population. Trade-offs between maximising health and promoting health equity are inevitable in this process. In this paper, we use the UK’s National Institute for Health and Care Excellence (NICE) as an example to examine how efforts to promote healthcare innovation in the priority-setting process can complicate these trade-offs. Drawing on NICE guidance, health technology assessment reports and relevant policy documents, we analyse under what conditions NICE recommends the National Health Service fund technologies of an “innovative nature”, even when these technologies do not satisfy NICE’s cost-effectiveness criteria. Our findings fail to assuage pre-existing concerns that NICE’s approach to appraising innovative technologies curtails its goals to promote health and health equity. They also reveal a lack of transparency and accountability regarding NICE’s treatment of innovative technologies, as well as raising additional concerns about equity. We conclude that further research needs to evaluate how NICE can promote health and health equity alongside healthcare innovation and draw some general lessons for healthcare priority-setting bodies like NICE.

## Background

In any health organisation with a limited budget, it is inevitable that the demand for healthcare outstrips the available resources. As new technologies are developed, decisions must be made about which to accommodate within the available budget. Some new technologies are rejected, despite their potential to offer benefits to particular patient groups. Other technologies are adopted, displacing resources that were previously assigned to other interventions and were benefitting other patient groups. Healthcare priority-setting aims to address the inevitable ethical trade-offs between maximising health and promoting health equity when health organisations choose to invest in some technologies over others (Sabik & Lie, 2008).

By determining the conditions under which novel technologies are funded, health organisations also influence private investment in future health technologies. A further aim of healthcare priority-setting is therefore sometimes to encourage investment and innovation (Ciani & Jommi, 2014). For example, the US Centers for Medicare and Medicaid Services (CMS) uses a “coverage with evidence development” approach to fund promising, but as yet unproven new treatments under the condition that patients participate in a registry or clinical trial (Tunis & Pearson, 2006; CMS, 2014). In the UK, new drugs funded through the Cancer Drugs Fund carry similar requirements for data collection (NICE, 2016a). Such approaches aim to promote healthcare innovation by reducing the financial risks associated with developing new treatments. But, depending on the given treatment, in doing so they may neither maximise health nor promote health equity.

To date, there has been little empirical analysis of how efforts to promote healthcare innovation through the priority-setting process might impact on the more familiar trade-off between maximising health and promoting health equity (Norheim et al., 2014). In this paper, we analyse a particular approach taken by the National Institute for Health and Care Excellence (NICE), the UK’s primary healthcare priority-setting body. We begin by providing some brief background on NICE and its approach to promoting innovation, which allows technologies of an “innovative nature” to be funded by the National Health Service (NHS) even when they do not satisfy NICE’s cost-effectiveness criteria. We then discuss pre-existing concerns that this approach curtails NICE’s goal to promote health and health equity in the NHS. By analysing relevant NICE documentation, we show that these concerns persist even with an in-depth understanding of the Institute’s policy and practice. Our findings reveal important insights into the challenges of promoting health and health equity alongside healthcare innovation in the priority-setting process.

## Innovation as a Value in Healthcare Priority-Setting?

In the UK, NICE plays an important role in setting healthcare priorities and deciding which new technologies the publicly funded NHS should adopt. Recognising that there is no consensus on how to set healthcare priorities, NICE has developed policies that combine both procedural and substantive criteria in one overarching health economic and ethical framework for health technology assessment (HTA) (NICE, 2008a).

This framework is best understood as a version of Norman Daniels’ “accountability for reasonableness” approach (AfR) (Daniels & Sabin, 2006). NICE’s framework emphasises the importance of a fair and deliberative priority-setting process, while setting out substantive health economic and ethical criteria that constrain the conditions under which NICE’s HTA committees might recommend technologies for funding by the NHS (Rid et al., 2015; Rumbold, Weale, Rid, Wilson, & Littlejohns, 2017). These criteria judge technologies primarily on their cost-effectiveness when compared with existing practice. Specifically, decisions are guided by calculating a technology’s “incremental cost-effectiveness ratio” (ICER): the financial cost of each additional quality-adjusted life year (QALY) offered by the technology when compared with the intervention currently in use (NICE, 2013a).

**Fig. 1:**
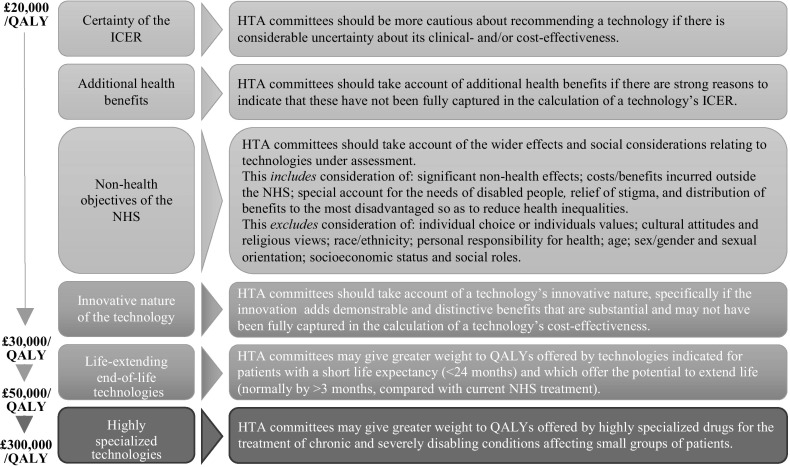
Factors recognised by NICE to justify funding technologies with an ICER > £20,000/QALY. Adapted from the 2013 Methods Guide, paras 6.3.3 and 6.2.10–11, by drawing on Social Value Judgements (2nd edition) and the 2017 Interim Methods Guide for the Highly Specialised Technologies programme (NICE, 2008a, 2013a, 2017a). A similar figure is included in NICE, 2013e

For a technology to be funded, NICE currently sets a cost-effectiveness threshold of £20,000–£30,000 per QALY gained. This threshold is intended to reflect “the opportunity cost of programmes that could be displaced by […] new technologies”, if a decision is made to fund them (NICE, 2013a). Technologies with ICERs < £20,000/QALY are considered to produce greater health benefits than the interventions they would displace and are generally recommended for funding based on cost-effectiveness alone. Within and beyond the threshold range, NICE’s framework allows other factors—including value-based considerations, or so-called social values—to justify funding some technologies, even when this decreases the total health that could be achieved with the available budget (Fig. 1) (Rawlins & Culyer, 2004; Rawlins, Barnett, & Stevens, 2010). For example, addressing the needs of disabled people, reducing health inequalities and providing life-extending treatments at the end of life are all social values explicitly recognised by NICE (NICE, 2008a, 2009, 2013a).

This framework not only ensures a transparent and relatively efficient use of limited NHS resources; by generally applying the same cost-effectiveness threshold across technologies and patient groups, it also establishes a default that gives all patients an equal claim on the available resources. At the same time, the framework recognises that social values can justify spending more on some patient groups than others. It thus promotes health and health equity as well as accountability (Rawlins & Culyer, 2004; Rawlins et al., 2010; Rid et al., 2015; Rumbold et al., 2017; Shah, Cookson, Culyer, & Littlejohns, 2013). As such, it is widely considered an exemplary approach to addressing ethical trade-offs in healthcare priority-setting.

A technology’s “innovative nature” is one of the social values that NICE advises its HTA committees to consider when evaluating technologies above the usual cost-effectiveness threshold. According to current policy, “if the innovation adds demonstrable and distinctive benefits of a substantial nature which may not have been adequately captured in the reference case QALY measure”, then a technology’s “innovativeness” can be invoked to support funding it at an ICER > £20,000/QALY (NICE, 2013a). However, NICE’s reasons for singling out innovative technologies in this way are unclear and, unlike most other social values, it is not intuitive why the innovative nature of a technology would justify its special treatment. The common-sense meaning of innovation—“different ways of doing things that bring improved outcomes” (Kennedy, 2009)—suggests that novelty is a means towards promoting health or health equity, and not an end in and of itself. Some have therefore argued that the innovative nature of a technology is not an independent social value, or, put differently, not a value in itself. Rather, its worth is derived from other social values, such as prioritising the severely ill, or from the value of the health gains that innovative technologies bring about (Bryan, Lee, & Mitton, 2013).

This possibility raises important concerns if innovation is used to support funding technologies above NICE’s usual cost-effectiveness threshold. If a technology’s innovative nature carries no independent value and NICE’s health economic and ethical framework already incorporates the values underpinning it—namely, promoting health or health equity—then funding technologies at the usual cost-effectiveness threshold would already reflect their full worth (McCabe, Claxton, & Culyer, 2008). Citing innovation to support funding technologies above the threshold would therefore be a spurious justification for spending limited resources on poorly cost-effective technologies. This would not only mean that more health could be achieved if the available funds were spent on more cost-effective interventions (McCabe et al., 2008). It would also mean that NHS patients have a legitimate complaint that some patient groups whose treatments are classified as innovative would receive more resources than they are entitled to. In other words, innovation, if it were spuriously treated as an independent social value, would curtail NICE’s goal of promoting health and health equity.

These concerns are heightened when one considers that, in practice, NICE already facilitates payment of a “premium” for novel technologies. All technologies appraised by NICE display some degree of novelty; for example, a drug may have been recently patented or an existing technology shown to be effective in a new application. This means that all technologies recommended by NICE are novel—or “innovative”—in some respect. NICE aims to ensure that recommended technologies are not overly costly relative to the interventions they displace by basing its cost-effectiveness threshold on an estimate of opportunity cost; that is, the health benefits that could have been achieved had the money been spent elsewhere in the NHS (NICE, 2013a). However, a recent study suggests that the mean cost of producing a QALY in the NHS is significantly less than NICE’s stated threshold of £20,000–£30,000/QALY—namely £12,936/QALY (Claxton et al., 2015). Moreover, in practice, NICE’s threshold for recommending technologies has been shown to be higher than this stated threshold (Dakin et al., 2015). Taken together, these studies suggest that NICE recommends novel technologies even when the cost per QALY is significantly higher than that of the interventions they are likely displacing. This is a notable “innovation premium” and presses the question whether an additional premium, in the form of innovation as an independent social value, is warranted. Financial rewards are, of course, important for incentivising healthcare innovation by private actors. Yet for healthcare priority-setters working with a limited budget, it is essential to consider when and by how much they should reward innovative technologies, and how their policies relate to other policies intended to promote innovation in health care, such as intellectual property arrangements (Kennedy, 2009; Green, 2010).

The concern that NICE’s policy on innovation curtails its goals of promoting health and health equity can only be evaluated with a sound understanding of how NICE defines innovation as a social value, and to what extent and how its HTA committees use it in practice. A literature search identified no empirical study of this topic to date (“Appendix 1”). The present paper therefore aims to address two questions. First, how often do NICE’s HTA committees invoke innovation to justify funding technologies that might otherwise be rejected as insufficiently cost-effective? If innovation is rarely used, this would alleviate concerns that special consideration of the innovative nature of novel technologies curtails the goal of promoting health and health equity. Second, does NICE’s policy define innovation, or do NICE’s HTA committees interpret this policy, in ways that clarify why and how innovation might be an independent social value? If the answer is yes and NICE’s policy or practice reveals a plausible conception of innovation, this would equally alleviate these concerns.

## Methods

We used a mixed methods approach to address these questions, combining quantitative and qualitative analysis of NICE’s policy documentation, publicly available HTA reports and other relevant policy reports.

### Analysis of NICE policy

To establish the evolution of NICE’s policy on innovation as a social value, we compiled a list of all NICE process and methods guides, addendums, amendments and social value documents published between the Institute’s inception in 1999 and June 2018 (available upon request). All 32 documents were systematically screened for the term “innov*” to identify relevant sections for analysis (“Appendix 2”). In addition, we analysed two reports on innovation commissioned by NICE (Kennedy, 2009; NICE Citizens Council, 2009) together with the Board’s response to these reports (NICE, 2010) and the current “user guide” for companies submitting evidence to NICE’s HTA process (NICE, 2015a).

### Quantitative analysis of NICE’s use of innovation

To explore how innovation has been invoked by NICE’s HTA committees, we performed a systematic analysis of the publicly available records of the “core” technology appraisal programme—NICE’s largest and longest established HTA workstream. We focused the analysis on drugs because these are almost all appraised via the core programme and have made up the majority of its HTAs, particularly in recent years. Between March 2000, when NICE’s first HTA was published, and the end of 2012, 218/270 HTAs (81%) related to drug products. This increased to 244/257 HTAs (95%) between January 2013, the year in which NICE’s current Methods Guide was released (thus marking the beginning of what we have approximately termed “current policy”), and June 2018, the cut-off date for the present analysis. Other technologies—such as medical devices, interventional procedures or diagnostic tools—are now predominantly appraised through smaller, specialised programmes and were therefore out of scope for our analysis (NICE, 2018a). Terminated HTAs that were never completed and HTAs that have been replaced or withdrawn were also excluded because relevant documentation was unavailable.

We established a comprehensive list of HTAs completed between March 2000 and June 2018 (based on NICE, 2018b; list available upon request) and accessed the webpage for each eligible HTA. We then retrieved the “final appraisal determination” (FAD), which sets out the rationale for the HTA committee’s final decision, and analysed this to determine how often, and how, innovation was invoked. Innovation was classed as having been not considered, not substantively considered (i.e. the word appeared in the FAD, but there was no evidence that innovation had been discussed by the committee in drawing its conclusions), or substantively considered. For those HTAs in which innovation was substantively considered, we also determined whether HTA committees judged the three conditions set out by NICE’s policy on innovation to have been met. (We did not to scrutinise whether the committees’ judgement seemed appropriate.) For these HTAs, we also recorded the committees’ funding recommendation and the ICER of the drug(s) concerned. Where ICERs were given as a range of plausible figures, we recorded the mid-point of the given range. For example, if the ICER was estimated at £20,900–£30,500/QALY, we recorded it as £25,700/QALY. Moreover, for drugs that were recommended at an ICER > £20,000/QALY, we determined whether innovation was invoked to justify this recommendation. All data were recorded and analysed by VC in Excel using simple descriptive statistics. AR spot checked a random sample of 26/527 (5%) FADs for accuracy and found no inaccuracies.

Additionally, during the quantitative analysis, we made brief free-text notes on HTA committees’ treatment of innovation for all FADs in which innovation was substantively considered. This allowed interesting interpretations of NICE’s policy on innovation to be identified.

### Qualitative analysis of NICE’s use of innovation

As part of a wider project on NICE’s social value judgements, three HTAs in which innovation appeared to have played a role in decision-making were purposively selected for in-depth qualitative analysis, utilising a systematic coding guide developed as part of the wider project (available on request). These HTAs were each completed during the “current policy” period, cited innovation as a contributory consideration in the FAD and related to drugs with ICERs > £20,000/QALY (i.e. exceeding the lower bound of NICE’s stated cost-effectiveness threshold). How HTA committees considered innovation did not influence sample selection; however, care was taken to ensure that the sample covered different conditions and included HTAs conducted by more than one of NICE’s four HTA committees. For each HTA, VC and AR read all publicly available documents and coded the FAD independently, resolving any disagreements by discussion. VC single-coded the remaining HTA documents relevant for this analysis (“Appendix 3”).

## Results

### NICE’s policy on innovation

NICE’s mandate to promote innovation dates back to the Institute’s creation in 1999. At this time, the government instructed NICE to guide the NHS’s uptake of new health technologies in ways that were “sympathetic to the longer-term interests of the NHS in encouraging innovation of good value to patients” (NICE, 1999; House of Commons Health Committee, 2002). By 2004, NICE itself referred more broadly to the need to encourage innovation “in technologies that will benefit patients” (NICE, 2004), subtly but markedly omitting the reference to innovation of good value. Also in the 2004 Methods Guide, NICE specified its cost-effectiveness threshold for the first time and introduced innovation as a social value, stipulating that “above a most plausible ICER of £20,000/QALY, judgements about the acceptability of the technology as an effective use of NHS resources are more likely to make more explicit reference to factors including […] the innovative nature of the technology” (NICE, 2004). Following debate as to whether NICE was doing enough to promote innovation (Cooksey, 2009; Green, 2010; Kennedy, 2009; NICE, 2010), this policy was strengthened. HTA committees are now instructed to “specifically take account of” a technology’s innovative nature at ICERs > £20,000/QALY (NICE, 2013a). This makes innovation one of only a handful of social values that NICE explicitly advises committees to invoke when recommending technologies that would otherwise be rejected as insufficiently cost-effective (Fig. 1).

Despite its long-standing commitment to promoting innovation, NICE has never clearly defined what has been described as a notoriously “amorphous concept” (Kennedy, 2009). Indeed, the instruction that HTA committees consider the innovative nature of a technology formed the entirety of the 2004 Methods Guide’s advice on the subject, and little further detail has been added since (Box 1). The current Methods Guide advises HTA committees to consider “if the innovation adds demonstrable and distinctive benefits of a substantial nature which may not have been adequately captured in the QALY measure” (NICE, 2013a). This wording is unchanged from the Guide’s previous version (NICE, 2008b) and is largely echoed in the current Social Value Judgements document, in which NICE articulates the ethical foundations of its work (NICE, 2008a). Elsewhere in the 2013 Methods Guide, NICE defines innovation more narrowly in terms of its potential to make a “significant or substantial impact on health-related benefits” (NICE, 2013a). The Manufacturers’ User Guide—aimed not at HTA committees but at manufacturers submitting evidence for assessment—also specifies that an innovative technology should offer significant health-related benefits; specifically, a “‘step-change’ in the management of the condition” under consideration (NICE, 2015a). NICE states in an earlier explanatory note that it is “for the Committee to decide […] what ‘step-change’ means” (NICE, 2010). The Institute does not provide any further guidance on its policy on innovation.

**Box 1:** NICE’s articulations of its policy on innovation as a social value

Guide to the methods of technology appraisal (3rd edition, Apr 2004): “Above a most plausible ICER of £20,000/QALY, judgements about the acceptability of the technology as an effective use of NHS resources are more likely to make more explicit reference to factors including […] the innovative nature of the technology” (NICE, 2004)
Guide to the methods of technology appraisal (4th edition, Jun 2008): “Above a most plausible ICER of £20,000 per QALY gained, judgements about the acceptability of the technology as an effective use of NHS resources will specifically take account of the following factors […] The innovative nature of the technology, specifically if the innovation adds demonstrable and distinctive benefits of a substantial nature which may not have been adequately captured in the QALY measure” (NICE, 2008b)
Social value judgements: Principles for the development of NICE guidance (2nd edition, Jul 2008): “Above a most plausible ICER of £20,000 per QALY gained, judgements about the acceptability of the intervention as an effective use of NHS resources will specifically take account of the following factors […] When the intervention is an innovation that adds demonstrable and distinct substantial benefits that may not have been adequately captured in the measurement of health gain” (NICE, 2008a)
Guide to the methods of technology appraisal (5th edition, Apr 2013): Section 2, “Developing the scope”: “Other issues likely to impact upon appraisal … the potential innovative nature of the technology, in particular its potential to make a significant and substantial impact on health-related benefits that are unlikely to be included in the QALY calculation during assessment” Section 6, “The appraisal of evidence and structured decision-making”: “Above a most plausible ICER of £20,000 per QALY gained, judgements about the acceptability of the technology as an effective use of NHS resources will specifically take account of the following factors […] The innovative nature of the technology, specifically if the innovation adds demonstrable and distinctive benefits of a substantial nature which may not have been adequately captured in the reference case QALY measure” (NICE, 2013a)
Single technology appraisal: User guide for company evidence submission template (Jan 2015) “If you consider the technology to be innovative, with potential to make a substantial impact on health-related benefits that are unlikely to be included in the quality-adjusted life year (QALY) calculation: state whether and how the technology is a ‘step-change’ in the management of the condition provide a rationale to support innovation, identifying and presenting the data you have used” (NICE, 2015a)

Considering the available guidance, we believe the best interpretation of NICE’s policy is that technologies must meet three conditions in order to qualify as innovative:

1. The technology must be of an “innovative nature” (NICE, 2013a) or display “innovative characteristics” (NICE, 2010) (novelty condition); *and*
2. The technology’s innovative characteristics must give rise to significant or substantial health-related benefits, also defined as a “‘step-change’ in the management of the condition” under consideration (NICE, 2015a) (substantial benefits condition); *and*
3. The substantial health-related benefits attributed to the innovative nature of the technology must be “demonstrable and distinctive” and not already captured in the technology’s ICER calculation (NICE, 2013a) (demonstrable and uncounted benefits condition). Presumably, this latter condition aims to prevent any double counting or factoring of unsubstantiated, speculative benefits.

When all three conditions are met, then HTA committees may invoke a technology’s innovative nature in recommending it at an ICER > £20,000/QALY.

### NICE’s consideration of innovation in practice

Between March 2000 and June 2018, NICE published 527 appraisals of technologies evaluated through its core HTA programme. Of these, 320/527 (61%) were included in the present analysis (Table 1).

**Table 1:** Analysis of drugs assessed through NICE’s core HTA programme (March 2000–June 2018)

	Full analysis period (Mar 2000–Jun 2018)	“Current policy” period^#^ (Jan 2013–Jun 2018)
All core HTAs	527/527 (100%)	257/527 (49%)
Included HTAs	320/527 (61%)	202/257 (79%)
Excluded HTAs*	207/527 (39%)	55/257 (21%)
Non-drug (e.g. medical devices, surgical interventions)	65/207 (32%)	13/55 (24%)
Terminated (i.e. never completed)	36/207 (17%)	24/55 (44%)
Obsolete (i.e. replaced or withdrawn)	106/207 (51%)	18/55 (32%)

^#^Period spanning the year in which NICE’s current Methods Guide was released and the cut-off for the present analysis

*Some appraisals could potentially have been excluded based on more than one of these criteria (e.g. an appraisal of a medical device may also have been terminated). In calculating these figures, exclusions were processed sequentially; that is, all non-drug appraisals were excluded first, then terminated appraisals and finally obsolete appraisals

**Fig. 2:**
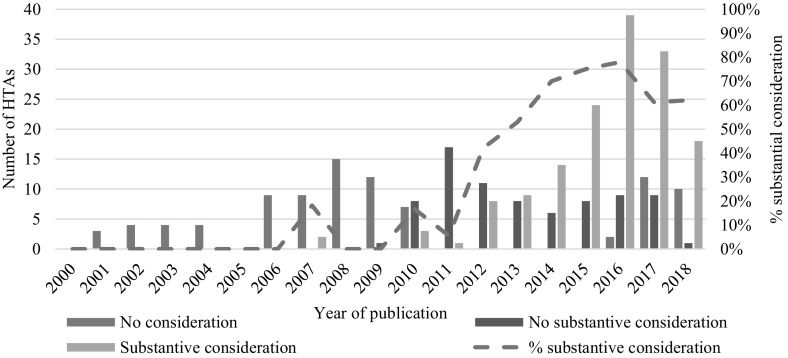
Consideration of innovation in NICE drug HTAs (March 2000–June 2018). Data are not included for HTAs completed in 2000 and 2005 as none met the criteria for inclusion in this study (i.e. non-obsolete, non-terminated drug appraisals)

In 151/320 (47%) in-scope HTAs, innovation was substantively considered, meaning that it was documented as having played a meaningful role in the HTA committee’s final deliberations (Fig. 2). The frequency with which innovation was substantively considered increased from 2010 onwards, following debate about NICE’s role in promoting innovation (Cooksey, 2009; Green, 2010; Kennedy, 2009; NICE, 2010). However, substantive consideration of innovation has only become a common feature of appraisals in recent years. Of the 151 HTAs substantively considering innovation, 137 (91%) were published after 2012.

**Fig. 3:**
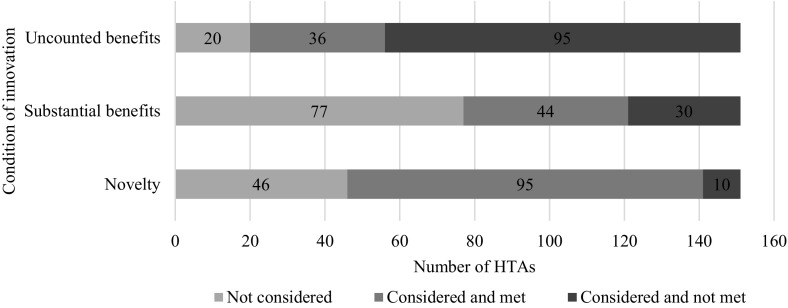
Consideration of NICE’s three conditions for innovation (March 2000–June 2018)

Across HTAs in which innovation was substantively considered, NICE’s HTA committees evaluated the demonstrable and uncounted benefits condition most consistently. Specifically, it was considered in 131/151 HTAs (87%) (Fig. 3). However, the demonstrable and uncounted benefits conditions were also the least likely to be judged to have been met, in 36/151 cases (24%). The novelty condition was considered in 105/151 cases (70%) and judged to have been met in 95/151 HTAs (63%). The substantive benefits condition was considered in 74/151 HTAs (49%) and was judged to have been met in 44 cases (29%).

**Fig. 4:**
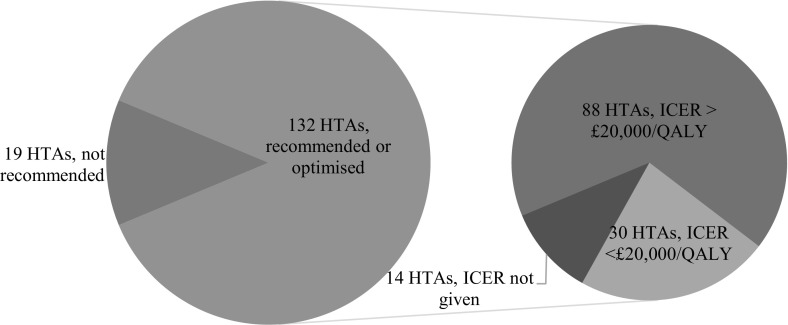
NICE drug HTAs in which innovation formed a substantive consideration, with recommendation for funding and estimated ICERs (March 2000–June 2018)

Of the 151 HTAs substantively considering innovation, 132/151 (87%) resulted in the drug being either fully recommended or recommended for use by specified patient groups (a so-called optimised recommendation). Eighty-eight of these 132 recommended technologies (67%) had estimated ICERs > £20,000/QALY, and 51/132 (39%) had estimated ICERs > £30,000/QALY (Fig. 4).

**Fig. 5:**
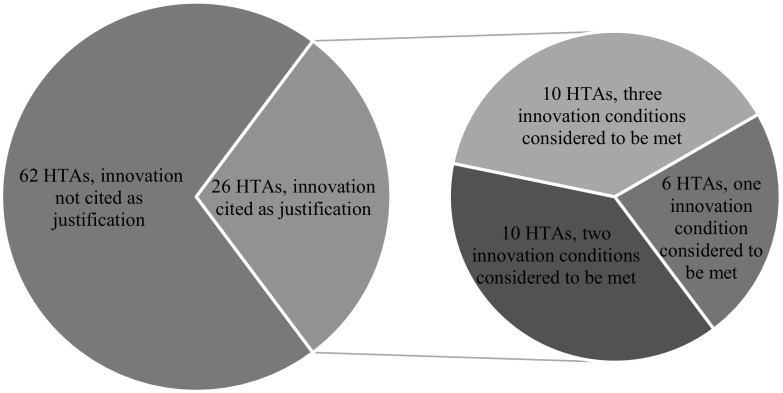
NICE HTAs in which drugs with ICERs > £20,000/QALY were recommended for funding (March 2000–June 2018). Innovation conditions considered to be met: HTA committees judged condition to be met; innovation conditions not considered to be met: HTA committees did not consider condition to be met or did consider condition and therefore failed to establish whether it was met

Of the 88 HTAs in which a drug was recommended for funding at an ICER > £20,000/QALY, 26 (30%) cited the drug’s innovative nature as at least partial justification for the recommendation (Fig. 4). In 10/26 HTAs (38%), committees considered the drug to meet all three conditions set out in NICE’s policy. In the remaining 16/26 HTAs (62%), committees invoked innovation to justify their funding recommendation without establishing that the three conditions of innovation were met or judging at least one of these conditions not to be met (Fig. 5). The condition most frequently considered to be met was the novelty condition: 23/26 (88%) technologies were thought to have innovative characteristics. The substantial benefits condition and the demonstrable and uncounted benefits condition were considered to be met in 17/26 (65%) and 16/26 (62%) HTAs, respectively. Of the 26 technologies recommended for funding, 14 (54%) had an ICER from £20,001 to £30,000/QALY, 8 (31%) had an ICER from £30,001 to £40,000/QALY, and 4 (15%) had an ICER from £40,001 to £50,000/QALY. Most of these technologies—19/26 (73%)—were appraised and recommended during the current policy period (i.e. since 2013).

### The meaning of innovation in practice

Three HTAs in which innovation was used to justify funding technologies with ICERs > £20,000/QALY were explored in more depth (Table 2).

**Table 2:** Committee judgements about innovation in selected HTAs in which innovation was cited to justify funding drugs with ICERs > £20,000/QALY (Jan 2013–June 2018)

	Case 1: TA388 (NICE, 2016b)	Case 2: TA297 (NICE, 2013b)	Case 3: TA282 (NICE, 2013c) & TA504 (NICE, 2018c)
Drug (brand name)	Sacubitril valsartan (Entresto)	Ocriplasmin (Jetrea)	Pirfenidone (Esbriet)
Target condition	Chronic heart failure	Vitreomacular traction	Idiopathic pulmonary fibrosis
Symptoms	Breathlessness, fatigue, swelling in the ankles/legs; eventually fatal	Decreased and distorted vision; sometimes loss of vision	Breathlessness, cough, fatigue, loss of appetite; eventually fatal
ICER (£/QALY)	Patient subgroup 1: 26,000 Patient subgroup 2: < 30,000	Patient subgroup 1: 20,900 Patient subgroup 2: < 30,500	24,000 (TA282) 25,000–29,000 (TA504)
Novelty condition	Considered met: “… the inhibition of neprilysin is a novel development in the pharmacological management of heart failure”	Considered met: provides an alternative to surgical intervention, which, though “effective in resolving vitreomacular traction”, carries risks and is “unpleasant” for patients	Considered met (TA282): “innovative mechanism of action” and “the first drug […] to have an impact […] in terms of improving outcomes without the long-term side-effects of immunosuppressants” Considered met (TA504): “reasonably innovative treatment at the time of the previous appraisal because it was the first drug to improve outcomes without the long-term side effects of immunosuppressants”
Substantial benefits condition	Considered met: offers a “small step-change in the management of this condition”	Considered met: offers a “step-change in treating patients with vitreomacular traction”	Considered not met (TA282): No “step-change” given the “modest treatment effect observed over a short duration” Not established (TA504)
Demonstrable and uncounted benefits condition	Not established	Considered not met: “… no significant or substantial health‑related benefits … that were not included in the economic model”	Considered not met (TA282): no “additional gains in health-related quality of life” excluded from economic model Considered not met (TA504): no “sizeable health-related benefits” excluded from the ICER
Other factors used to justify the drug’s “innovativeness”	Historical underinvestment in the disease area Promising Innovative Medicine (PIM) designation granted by MHRA.	None	“Disease of high unmet need” (TA282). Precedent (TA504): “the committee understood that pirfenidone was considered a reasonably innovative treatment at the time of the previous appraisal”.
Other values invoked to justify funding the drug	None	None	“Acceptable level of uncertainty … within the range normally considered to represent a cost-effective use of NHS resources” (TA282). Precedent/existing access to treatment (TA504): the committee concluded that “it had not seen any clinical evidence contradictory to” that previously considered and was “not minded to change this recommendation and withdraw an existing treatment option for people with moderate disease, despite the relatively high ICERs”
Funding recommendation	Recommended for both subgroups	Recommended for both subgroups	Recommended in both appraisals

Case 1 concerned sacubitril valsartan, a new treatment for chronic heart failure. The HTA committee recommended funding sacubitril valsartan at an estimated ICER of £26,000–£30,000/QALY “given its innovative nature” (NICE, 2016b). It cited no other social values to support this recommendation. The committee considered the drug to meet NICE’s novelty condition, given its novel mode of biological action. The committee also considered the drug to meet the substantial benefits condition because sacubitril valsartan offers “a small step-change in the management of this condition”, even though the committee recognised “considerable uncertainties” about the drug’s health-related benefits. Moreover, these benefits were found to be “not statistically significant” in the Western European subgroup most relevant to UK practice (NICE, 2016b). The committee did not make any reference to the demonstrable and uncounted benefits condition. However, it invoked several other value-based considerations to support its treatment of sacubitril valsartan as innovative, including the unmet need of patients living with chronic heart failure, historical under-investment in the disease area and the fact that the UK Medicines and Healthcare Products Regulatory Agency (MHRA) had granted the drug a Promising Innovative Medicine (PIM) designation (NICE, 2016b).

In Case 2, the HTA committee recommended funding ocriplasmin—an injectable drug to treat a sight-affecting eye condition—for two subgroups of patients at estimated ICERs of £20,900/QALY and < £30,500/QALY (NICE, 2013b). Ocriplasmin’s innovative nature was the only social value invoked in support of this recommendation. The committee considered ocriplasmin to meet the novelty condition because the drug offers a less invasive alternative to the standard treatment of “watch and wait” followed by surgical intervention as needed. Specifically, the committee noted that while surgery is “effective”, it entails risks and an “unpleasant” recovery for patients (NICE, 2013b). The committee also considered ocriplasmin to offer a “step change [sic]” in treating patients and hence fulfil the substantial benefits condition. However, it identified “no significant or substantial health-related benefits… that were not included in the economic model [ICER]” (NICE, 2013b), meaning that it did not consider the demonstrable and uncounted benefits condition to be met.

Case 3 involved the appraisal and reappraisal of pirfenidone—a drug for treating idiopathic pulmonary fibrosis—by two separate HTA committees. The first committee recommended pirfenidone for funding at an estimated ICER of £24,000/QALY (NICE, 2013c). It judged pirfenidone to meet the novelty condition because of its “innovative mechanism of action”, and because it is the “first drug” to improve outcomes without the long-term side effects of immunosuppressants for a condition of “high unmet need” (NICE, 2013c). However, given pirfenidone’s “modest effect observed over a short duration”, the committee did not consider the substantial benefits condition to be fulfilled. Furthermore, the committee identified “no additional QALYs that had not been incorporated into the economic model” (NICE, 2013c), meaning that the demonstrable and uncounted benefits condition was not met. It therefore did not invoke innovation to justify funding pirfenidone, instead citing the “acceptable level of uncertainty” associated with the ICER and the fact that this fell “within the range normally considered to represent a cost-effective use of NHS resources” (NICE, 2013c).

In a reappraisal triggered by new clinical trial data, a second HTA committee maintained the recommendation to fund pirfenidone at an updated ICER of £25,000–£29,000/QALY (NICE, 2018c). Yet unlike the first committee, it justified this recommendation partly based on innovation. It noted that the drug had been “considered a reasonably innovative treatment at the time of the previous appraisal” and, given that “it had not seen any evidence contradictory to that considered in NICE’s… previous appraisal…, it was not minded to… withdraw an existing treatment option” (NICE, 2018c). The committee did not mention the substantial benefits condition and still considered the demonstrable and uncounted benefits condition to be unfulfilled. Thus, the judgement that pirfenidone met the novelty condition supported its justification to recommend its continued funding.

These in-depth case studies, together with other examples identified through the quantitative analysis, also reveal variation in how HTA committees interpret the three conditions set out by NICE (Box 2). In relation to the novelty condition, HTA committees differed in both the type and magnitude of innovation they considered necessary to fulfil it. Some committees designated a whole drug class as novel, such as disease-modifying anti-rheumatic drugs (NICE, 2016c). Other committees only considered “first-in-class” drugs to meet the novelty condition, notwithstanding the “pharmacological progress” offered by subsequent products, such as second-generation tyrosine kinase inhibitors for treating leukaemia (NICE, 2016d). Yet other committees differed on whether health benefits were sufficient evidence of innovation. For example, one committee considered a monoclonal antibody for treating cancer to be novel because it had “low toxicity and [a] favourable adverse effects profile”, even though the antibody had no “unique” mechanism of action (NICE, 2016e). Another committee did not consider a different antibody to be innovative because it “did not differ substantially in its mechanism of action” from existing treatments, its health benefits for the given indication notwithstanding (NICE, 2017b). Committees also varied in their willingness to attribute novelty to long-established drugs used slightly differently within an existing indication or for a new indication. One committee, for example, considered a well-established cancer drug to be novel when used earlier than usual in the treatment pathway (NICE, 2016f). By contrast, a different committee made the opposite judgement about another well-established cancer drug when used later than usual in the treatment pathway (NICE, 2014a).

**Box 2:** Examples of variation in HTA committees’ interpretations of the conditions for innovation set out by NICE policy

*Novelty condition*
Novelty of classes of drugs
In TA426, the HTA committee stated that although “second-generation” tyrosine kinase inhibitors represented an “important development in terms of pharmacological progress”, the “critical innovation” had been the development of the “first-in-class” drug (NICE, 2016d). In contrast, in TA375 the committee concluded that “the biological DMARDs [disease-modifying anti-rheumatic drugs] should be considered an innovative class of drugs” despite several of its members being “second-generation” DMARDs (Kristensen et al., 2011; NICE, 2016c)
Health benefits as *ipso facto* evidence of novelty
In TA384, the committee acknowledged that the cancer drug nivolumab’s mechanism of action was not “unique”, but accepted its “low toxicity” and “favourable adverse effect profile” alone as evidence of innovation (NICE, 2016e). Ixekizumab, appraised in TA442, also offered health benefits, but the committee noted that it “did not differ substantially in its mechanism of action” from existing treatments and did not class it as innovative (NICE, 2017b)
Novel use within an existing indication
In TA387, the committee acknowledged that abiraterone was “not a new” prostate cancer drug, but concluded that it could be considered innovative because “it was the first active treatment available for this position in the treatment pathway” (NICE, 2016f). In contrast, in TA326 the committee stated that although using imatinib at a novel stage in the treatment of gastrointestinal stromal tumours could offer benefits, it had “been available as a treatment […] for many years” and consequently could not be considered innovative (NICE, 2014a)
Novelty of developing an existing drug for a new indication
In TA300, the committee recalled that three anti-viral therapies for hepatitis C “were likely to have been innovative” when first used in adults, but judged that they could “no longer be considered innovative” when later used in children (NICE, 2013d). In contrast, committees have judged nivolumab to be innovative for several cancer types (e.g. renal cell carcinoma (NICE, 2016g), Hodgkin lymphoma (NICE, 2017c) and lung cancer (NICE, 2017d)), despite one acknowledging that “it was not the first checkpoint inhibitor to gain a marketing authorisation for treating cancer” (NICE, 2016g)
Novelty after many years of established use
In TA369, the committee concluded that ciclosporin—first licensed in 1983—“was not a novel technology”, despite its novel formulation (NICE, 2015g). In contrast, in TA373 the committee concluded that although the drugs in question had “been in use for a long time” (since the late 1990s), they “remain a step‑change in the management of JIA [juvenile idiopathic arthritis] […] and, as such, are innovative” (NICE, 2015b)
*Substantial benefits condition*
Magnitude of substantial benefits
In TA388, the committee concluded that sacubitril valsartan offered a “small step-change” in patient outcomes for chronic heart failure, despite “non-significant results” in the most relevant trial population (NICE, 2016b). In contrast, in TA282 the committee concluded that pirfenidone’s “modest [treatment] effect” meant that it could not be considered “a step change [sic] in the management of idiopathic pulmonary fibrosis” (NICE, 2013c)
Psychological benefits
In TA358, the committee recognised the “positive psychological benefit” that patients suffering from a genetic kidney disorder would derive from having an additional option for treatment of a condition with high unmet need (NICE, 2015c). In contrast, in TA403 the committee concluded that “having an extra treatment option” available in another area of high unmet need—advanced non-small-cell lung cancer—did not constitute a relevant benefit (NICE, 2016h)
Indirect health benefits
In TA269, the committee acknowledged that the development of the mutation-specific TKI vemurafenib had “advanced the understanding” of malignant melanoma and took this into account in recommending the drug (NICE, 2012a). In contrast, in TA259 the committee judged that “health benefits likely to accrue” from publicly funded research financed by sales of abiraterone fell “outside of NICE’s policy regarding innovation” (NICE, 2012b)
Non-health benefits
In TA363, the committee recognised the “improved earning capacity” of hepatitis C patients receiving treatment with ledipasvir–sofosbuvir and recognised the reduction in ICER this would bring about (NICE, 2015d). In contrast, in TA415 the committee rejected the manufacturer’s claim that certolizumab pegol’s effect “on workplace and household productivity” should be taken into account in adjusting the technology’s likely ICER (NICE, 2016i)
*Demonstrable and uncounted benefits condition*
Demonstrability of benefits
In TA388, the committee judged sacubitril valsartan to constitute a “step-change” in the treatment of heart failure despite “considerable uncertainties” about the magnitude of its benefits (NICE, 2016b). In contrast, in TA282, the committee considered that pirfenidone could not be considered a “step change [sic]” treatment for pulmonary fibrosis, in part because its apparent benefits had only been observed over “short duration” (NICE, 2013c)

In considering the substantial benefits condition, HTA committees similarly differed in the types and magnitude of benefits they considered necessary to fulfil it. For example, the committee appraising sacubitril valsartan (discussed above) accepted relatively insubstantial benefits as a “small step-change” in the management of the condition (NICE, 2016b). By contrast, the committee appraising pirfenidone (also discussed above) did not consider a “modest [treatment] effect” to be a substantial benefit (NICE, 2013c, 2018c). Committees also differed in the types of benefits they recognised as relevant. In judging whether a drug was innovative, some considered only direct health-related benefits. They rejected claims, for example, that improvements in “workplace and household productivity” from a rheumatoid arthritis drug should be taken into account (NICE, 2016i). By contrast, other committees factored indirect and non-health benefits into their consideration of a drug’s innovativeness, against NICE’s current policy. For instance, several committees took account of the “wider benefits to society”, including reduced disease transmission and patients’ improved earning capacity, in deciding to treat novel hepatitis C drugs as innovative (NICE, 2015d, e, f). Another committee considered it a benefit that a novel cancer drug “had advanced the understanding” of a particular disease area and “opened the way to new treatments”. The committee cited the “combined value” of this and other considerations, including special consideration of a life-extending treatment at the end of life, to support its recommendation for funding the drug (NICE, 2012a). Finally, HTA committees took different stances on the relevance of psychological benefits. For example, when assessing drugs for conditions with high unmet need, one committee factored the “positive psychological benefit” derived from having an additional treatment option, while another concluded that this type of benefit should not be taken into account (NICE, 2015c, 2016h).

HTA committees also differed in their consideration of the demonstrable and uncounted benefits condition. For example, the first committee to appraise pirfenidone (discussed above) considered this condition not to be met because the benefits attributable to its innovative nature were too uncertain (NICE, 2013c). By contrast, the committee appraising sacubitril valsartan (also discussed above) considered the drug to meet the condition despite “considerable uncertainties in the data” (NICE, 2016b). Moreover, several committees that classified technologies as innovative acknowledged that the demonstrable and uncounted benefits condition was not fulfilled. For example, the committee appraising ocriplasmin found “no significant or substantial health-related benefits […] that were not included in the economic model”, but still recommended the drug for funding at an ICER > £20,000/QALY because it considered it to be innovative (NICE, 2013b).

Finally, when justifying a drug’s innovative nature, HTA committees invoked considerations beyond those set out by NICE’s policy. Returning to our three case studies, “historical under-investment” in the disease area and “high unmet need” contributed to both sacubitril valsartan and pirfenidone being treated as innovative (NICE, 2013c, 2016b). The committee appraising sacubitril valsartan also noted precedents set by other bodies, such as the MHRA’s decision to grant it a PIM designation—even though the criteria for innovation according to this scheme differ from NICE’s conditions of innovation (Medicines and Healthcare products Regulatory Agency, 2014; NICE, 2016b). Similar precedent-based arguments were also made in other HTAs (NICE, 2017c, d).

## Discussion

Special consideration for “innovation” is anchored in NICE’s statutory origins and has been an explicit part of its HTA policy since 2004. However, to date it has not been clear exactly what NICE’s policy on innovation is, and when and how committees invoke innovation to justify recommending technologies that might otherwise be considered insufficiently cost-effective. This has made it difficult to evaluate important concerns that innovation is not an independent social value—that is, that it is not a value in and of itself, but is derived from the more fundamental values of promoting health or health equity—and that its use therefore curtails NICE’s goals of promoting health and health equity in the NHS (Bryan et al., 2013; McCabe et al., 2008). Our study, the first systematic empirical analysis of NICE’s policy and practice regarding innovation as a social value, attempts to address this research gap.

Our results show, first, that special consideration of innovation appears to play an increasingly important role in how NICE sets healthcare priorities for the NHS. Before 2013, HTA committees substantively considered innovation in only a small percentage (12%) of HTAs. This percentage rose to 68% between 2013 and 2018, despite NICE’s policy on innovation remaining formally unchanged. When committees considered innovation and recommended funding a drug above NICE’s stated cost-effectiveness threshold, they used innovation to at least partially justify their recommendation in a significant minority of cases (30% or 26/88 HTAs). Moreover, in some of these cases committees appeared to treat innovation as an independent social value. For example, the committee that appraised sacubitril valsartan concluded that an ICER “at the upper end” of NICE’s stated cost-effectiveness threshold could be considered acceptable “*given its innovative nature*” [emphasis added], even though it considered sacubitril valsartan to offer only a “small step-change” in managing heart failure, identified no uncounted health-related benefits and invoked no other social values in support of this decision (NICE, 2016b).

Our analysis does not allow us to determine whether innovation was decisive in causing HTA committees to recommend technologies with ICERs > £20,000/QALY, particularly where other social values (e.g. special consideration of life-extending treatment at the end of life) were taken into account. On average, committees have been shown to recommend technologies well above the stated £20,000–£30,000/QALY threshold (Dakin et al., 2015). It is therefore possible that the technologies we identify as having been recommended at least in part due to their “innovativeness” would have been recommended regardless. It is also possible that some committees do not support innovation as an independent social value, but invoke it for other reasons, for example, to bolster the case for recommending a particular technology which is supported by other social values, or to appear compliant with NICE policy on innovation. Further research is needed to examine these possibilities. However, we note that in two of the three HTAs selected for in-depth analysis, innovation was the only social value that committees invoked to support funding technologies > £20,000/QALY. Moreover, in each of these three cases innovation was cited in the key paragraph describing the committees’ rationale for recommending the drug, suggesting that it did at least feature in the decisive discussion. In addition, almost half of the 26 recommended technologies in which innovation was cited as justification had ICERs > £30,000/QALY. This suggests that even if NICE’s de facto threshold considerably exceeds the stated figure of £20,000–£30,000/QALY (Dakin et al., 2015), innovation may be playing a role in supporting recommendations that cannot easily be justified on the basis of cost-effectiveness alone. Pending more conclusive research on how innovation influences NICE’s final recommendations, our analysis clearly demonstrates its growing importance in the Institute’s deliberations. Concerns about innovation as a social value therefore cannot be dismissed as irrelevant for current practice.

Second, our analysis of NICE’s policy and HTA committee decisions sheds little light on why or how a technology’s innovative nature might be an independent social value, or a value in itself. NICE’s policy sets out three conditions for innovation: the novelty condition, which requires that the technology under consideration must have innovative characteristics; the substantial benefits condition, which requires that these characteristics lead to substantial health-related benefits; and the demonstrable and uncounted benefits condition, which requires that the benefits be supported by adequate evidence and not already be part of the standard ICER calculation, thereby preventing double counting. The novelty and substantial benefits conditions reflect the common-sense understanding of innovation: “different ways of doing things that bring improved outcomes” (Kennedy, 2009). This brings us back to the concern that motivated this paper; namely, that innovative technologies might not be independently valuable. The two conditions clarify that NICE values innovative technologies insofar as they are novel and lead to substantial health-related benefits or promote health equity. However, they provide no indication of why innovative technologies should be valued above and beyond their health- or equity-related benefits. For example, NICE’s current policy does not indicate why innovative technologies might be worthy of greater investment than other technologies that lead to comparable levels of health benefit. The demonstrable and uncounted benefits condition does not clarify the issue, as it simply asks committees to consider whether the given ICER adequately captures all health-related benefits—which is a consideration for all technologies with an ICER > £20,000/QALY, innovative or not (NICE, 2013a).

Our analysis of how HTA committees interpret NICE’s policy does offer some interesting insights into why innovative technologies might be valued over and above their health-related benefits. For example, one committee considered it a benefit that a novel cancer drug had “opened the way to new treatments” (NICE, 2012b). This suggests that innovative technologies might be especially valuable because they lay the foundation for other important healthcare innovations, as suggested elsewhere in the literature (Garner, 2010; Henshall and Schuller, 2013; Kennedy, 2009; NICE Citizens Council, 2009). However, as we discuss in the following paragraphs, committees did not have a shared understanding of NICE’s policy that might have revealed a plausible working definition of innovation as a social value. Thus, neither NICE’s policy nor its interpretation by HTA committees helps to illuminate why or how innovation might be valuable in and of itself. Concerns that innovation is a spurious social value whose use leads to health loss and inequity in the NHS therefore still stand (Bryan et al., 2013; McCabe et al., 2008).

Third, our analysis finds that NICE’s policy on innovation is vague and implemented in diverse—and sometimes conflicting or problematic—ways. NICE devotes only a few lines of its 94-page 2013 Methods Guide to defining innovative technologies and specifying how they should be treated (NICE, 2013a). Moreover, aspects of the policy are currently spread across several documents (NICE, 2008a, 2010, 2013a, 2015a) and require considerable interpretation. The three conditions that, in our view, make up the best rendering of NICE’s policy on innovation are barely specified. In fact, the novelty condition is not specified at all—although proposed definitions of innovation or novelty, especially in the context of drug development, have existed for years (Aronson, Ferner, & Hughes, 2012; Ciani et al., 2016; De Sola-Morales et al., 2018; Ferner, Hughes, & Aronson, 2010; Kesselheim, Wang, Avorn, 2013). Nor is the novelty condition explicitly set out. An uncharitable interpretation of NICE’s policy therefore suggests—implausibly in our view—that an innovative technology need not be novel, so long as it yields substantial and previously uncounted benefits (NICE, 2013a).

Given these ambiguities, it is not surprising that NICE’s HTA committees have implemented the policy in diverse ways. First, a significant proportion of committees considered technologies to be innovative even when they did not establish that NICE’s three conditions for innovation were met. In addition, HTA committees applied a wide range of interpretations of these conditions, some of which stood in direct conflict. For instance, some committees invoked wider benefits to society, such as patients’ improved earning capacity, to satisfy the substantial benefits condition. Others explicitly rejected taking such benefits into account. Committees also interpreted NICE’s policy in ways that seem difficult to justify. For example, some considered a whole drug class to meet the novelty condition, even though this would—implausibly—imply that “me-too” drugs or other technologies lacking in significant pharmacological innovation deserve special treatment due to their “innovativeness”. Perhaps of most concern, a considerable number of HTAs judged a drug to be innovative based on its ability to offer health benefits, without demonstrating any novelty.

Taken together, these findings strengthen concerns about NICE’s treatment of innovation. The ambiguities at the level of policy mean that NICE’s stakeholders, and notably patients who are negatively affected by its funding recommendations, are not able to follow how and why some technologies are judged to be innovative and therefore deserve special consideration. Yet being transparent about how and why technologies are—or are not—recommended for funding, and grounding recommendations in reasons that all can accept as relevant to setting healthcare priorities, are essential to the AfR approach that NICE espouses (Daniels, 2000; Daniels & Sabin, 2006; NICE, 2008a). As others have pointed out, clear definitions of terms such as a technology’s “innovative nature” are particularly important for ensuring transparency and accountability (Ferner et al., 2010; Green, 2010; Kennedy, 2009). Our analysis shows that such definitions are mostly lacking and hence raises concerns that NICE is not sufficiently accountable regarding how it uses one of its key social values. Indeed, even if HTA committees did not actually treat innovation as a key social value, concerns about accountability would remain as long as they cite innovation as part of their rationale in public HTA reports.

In addition, the significant variation in how HTA committees interpreted NICE’s policy on innovation raises concern that relevantly similar patients are not being treated equally. A key principle of justice is that of formal equality—that is, the principle that cases should be treated alike when they are as like in relevant respects (Gosepath, 2011). But if committees are differing significantly in their treatment of innovative technologies as our analysis suggests, it seems highly probable that cases that are alike in relevant respects are not being treated as like. One would, of course, expect some variation in how committees interpret NICE’s policy—any policy, however well specified, requires interpretation. One could also argue that, as long as funding recommendations are made through a fair and transparent process, the variation in committees’ interpretations is no cause for concern. However, the AfR framework that NICE espouses does not take a purely procedural approach to justice, according to which there is no independent criterion for the just or fair outcome (Rid, 2009). Instead, NICE’s AfR framework is committed to health economic and ethical criteria that constrain the conditions under which HTA committees might recommend new technologies (Rid et al., 2015; Rumbold et al., 2017). For example, although the extent to which wider societal benefits should be factored in healthcare priority-setting is debated (Brock, 2003; Culyer et al., 2018; Du Toit & Millum, 2016; Linley & Hughes, 2013; NICE Citizens Council, 2008; Miners, A., Cairns, J., & Wailoo, 2013; Shearer, Byford, & Birch, 2017), NICE explicitly excludes some of these benefits, such as economic productivity, from its health economic calculations (NICE, 2013a). Our finding that some HTA committees still cite patients’ improved earning capacity in judging technologies as innovative (NICE, 2015d, e, f) therefore suggests at least some unwarranted variation in how committees interpret NICE’s policy on innovation. The same likely applies to other identified problematic interpretations of NICE’s policy, such as entire classes of drugs being classified as novel or innovative.

Needless to say, there is nothing unique about the concern that NICE is insufficiently accountable regarding its use of innovation as a social value and that HTA committees vary unduly in some of their funding recommendations. Any social value that is ambiguous will raise similar concerns. Indeed, some of the other social values that NICE recognises are less specified than innovation. For example, NICE endorses the value of “reducing health inequalities” and identifies “offering particular benefit to the most disadvantaged” as one way of realising this value (NICE, 2008a). However, the Institute provides no further guidance on how the most disadvantaged should be identified for the purpose of setting healthcare priorities, even though this is a complex question (Sharp & Millum, 2018). This means that NICE’s policy on health inequalities would likely raise similar concerns about accountability and unequal treatment as NICE’s policy on innovation. At the same time, NICE has specified other social values to a greater extent than innovation. For example, NICE uses detailed criteria to specify the value of giving special consideration to life-extending treatment at the end of life and lifts its stated cost-effectiveness threshold for these treatments by a specified amount: to £50,000/QALY (NICE, 2009, 2013a, 2016a). While providing very detailed guidance can raise its own concerns—for example, about limiting deliberation and critical judgement—our findings suggest that NICE should provide more detail on the value of innovation in order to set reasonable bounds to how HTA committees interpret it in practice. Given this and the continued concern that NICE’s use of innovation as a social value might curtail health and health equity among NHS patients, we urge the Institute to review its current policy on innovative technologies.

### Limitations and future work

Our analysis is limited to drugs assessed through NICE’s core technology appraisal programme; we did not examine how innovation is treated in other programmes or for other technology types. While expanding our analysis might offer further insights, the core programme is NICE’s largest and most long-established HTA programme. Moreover, it is one of only two programmes—the other being a small programme dedicated to highly specialised technologies—whose recommendations carry an NHS funding mandate. Our analysis thus provides a comprehensive overview of NICE’s most important and most influential HTA activity.

Our analysis is based exclusively on HTA documentation, which—though detailed—is unlikely to reflect HTA committees’ deliberations in their entirety. In particular, it is possible that committees substantively considered conditions for innovation without this being documented. Their implementation of NICE’s policy on innovation may therefore have been more complete than our data suggest. It is also possible that committees gave more—or less—weight to a technology’s innovative nature than the HTA reports convey. Future studies should address these uncertainties, for example by interviewing HTA committee members about selected past recommendations.

Our in-depth analysis of how HTA committees understood innovation covered only a very small proportion of HTAs: 3/151 HTAs (2%) in which committees substantively considered innovation and 3/26 HTAs (12%) in which they used innovation to at least partly justify recommending technologies > £20,000/QALY. In addition, this analysis focused by chance on HTAs in which the committees did not consider NICE’s three conditions for innovation to be met. It also did not include cases in which technologies may have satisfied these conditions but were inappropriately judged not to have met them. Future studies should expand the in-depth analysis accordingly.

Due to ambiguities in NICE’s policy on innovation, our analysis rests on a particular interpretation. We have aimed to adopt the most charitable approach to interpreting this policy and examining its implementation in practice; for example, we did not question whether HTA committees appropriately judged NICE’s conditions for innovation to be met. Future studies should explore whether our interpretation of NICE’s policy on innovation is accurate, for example by interviewing relevant NICE staff. Future work might also examine whether committees appropriately apply NICE’s conditions for innovation, for instance by surveying members of its HTA committees and asking them to apply them to purposely designed vignettes.

Finally, our analysis is not designed to address whether NICE or other HTA bodies are justified in treating innovation as an independent social value. Our examination of NICE’s policy and practice did not offer any compelling evidence to support such use, but legitimate arguments in support of innovation as an independent social value might still exist. Future research should explore why NICE (and perhaps other HTA bodies) recognises innovation as a value, for example by interviewing relevant staff and HTA committee members. It should also examine whether their views withstand ethical scrutiny, for instance by analysing to what extent their arguments stand for themselves or mirror already recognised considerations of health equity, such as disease severity or past health loss given a high unmet need for treatment (Norheim et al., 2014). Health economic or ethical analysis might also reveal arguments that explain or justify why innovation is valuable in and of itself.

### International implications

While our study focuses on NICE—a single healthcare priority-setting body offering advice in a single jurisdiction—our analysis is of interest well beyond the UK. How to use HTA in a way that stimulates innovation—or, more precisely, the right kind of innovation—is a challenge that all healthcare priority-setting bodies face, their methodological and organisational differences notwithstanding (Henshall & Schuller, 2013). Moreover, NICE is not alone in using innovation as an evaluative criterion (Angelis, Lange, & Kanvos, 2018); for example, the Italian Medicine Agency recently issued guidance on when to classify drugs as innovative (AIFA, 2017). Yet to date, there are no empirical studies of how healthcare priority-setting bodies use innovation as a criterion, and how it might impact on health and health equity. The challenges that NICE faces in terms of identifying and responding to the unique value of innovative technologies are ones that any body like NICE needs to address when it aims to promote innovation through healthcare priority-setting. Moreover, NICE is often regarded as a “forerunner” in integrating social value judgements into its methodology and serves as an example for healthcare priority-setting bodies internationally (Angelis et al., 2018). Finally, UK drug prices—which are shaped by NICE’s funding recommendations—are also known to inform price negotiations around the world through the practice of international reference pricing (Office of Fair Trading, 2007). As such, any “premium” paid for innovative drugs in the UK may have repercussions for health and health equity elsewhere.

## Appendix 1: Literature search

**Search terms:**

Title/abstract/key (drug OR pharmaceutical OR medicine OR medication OR “health technology” OR “medical technology” OR treatment OR therapy)

AND

Title/abstract/key (“technology assessment” OR “technology appraisal” OR HTA OR “cost–benefit analysis” OR “cost effectiveness” OR “comparative effectiveness research” OR “economic evaluation” OR “healthcare rationing” OR “health care rationing” OR “health policy” OR “healthcare policy” OR “health care policy” OR “resource allocation” OR “health care allocation” OR “healthcare allocation” OR “health care coverage” OR “healthcare coverage” OR “health priorities” OR “priority setting” OR “health technology prioritisation” OR “health technology prioritization” OR “National Institute for Clinical Excellence” OR “National Institute for Care Excellence” OR “National Institute for Health and Care Excellence”)

AND

Title/abstract/key (“social value” OR “social norm” OR “societal value” OR “moral value” OR “moral norm” OR “value judgement” OR “value judgment” OR equity OR fairness OR fair OR justice OR “trade off” OR ethic* OR “human right” OR “right to health”)

AND

Title (innov*)

AND

Language (English)

**Databases:** PubMed; Scopus; ProQuest; Web of Science

**Time frame:** No upper or lower limit

**Total number of identified papers:** 143 (excluding duplicates)

**Number of relevant papers:** 9 (based on title and/or abstract screening)

**Independently identified papers**: 9

## Appendix 2: Documents included in systematic review of NICE policy

**Table:**

Year	Key policy documents*	Supporting documents
1999	Appraisal of new and existing technologies: Interim guidance for manufacturers and sponsors	
2001	Guide to the technology appraisal process (1st ed.) Guidance for manufacturers and sponsors/Guide to the methods of technology appraisal (1st ed.)^#^ Guidance for appellants	Guidance for healthcare professional groups Guidance for patient/carer groups
2004	Guide to the methods of technology appraisal (2nd ed.) Guide to the technology appraisal process (2nd ed.) Technology appraisal process: guidance for appellants	A guide for manufacturers and sponsors A guide for healthcare professional groups A guide for NHS organisations A guide for patient/carer groups
2005	Social value judgements: Principles for the development of NICE guidance (1st ed.)	
2006	Guide to the single technology appraisal process (1st ed.)	
2007		Single Technology Appraisal Process: update
2008	Guide to the methods of technology appraisal (3rd ed.) Social value judgements: Principles for the development of NICE guidance (2nd ed.)	
2009	Guide to the single technology appraisal process (2nd ed.) Guide to the multiple technology appraisal process (3rd ed.)	Supplementary advice: appraising life-extending, end-of-life treatments
2010	Appeals process guide	
2011		Clarification on discounting
2013	Guide to the methods of technology appraisal (4th ed.)	
2014	Guide to the processes of technology appraisal (4th ed.) Guide to the technology appraisal and highly specialised technologies appeal process	
2016		Addendum A—final amendments to the NICE technology appraisal processes and methods guides to support the proposed new Cancer Drugs Fund arrangements Rapid reconsideration of drugs currently funded through the Cancer Drugs Fund
2017		Fast track appraisal: addendum to the Guide to the processes of technology appraisal Cost comparison: addendum to the Guide to the processes of technology appraisal Procedure for varying the funding requirement to take account of net budget impact
2018	Guide to the processes of technology appraisal (4th ed.)—2018 update	

*Key policy documents include the methods guides, process guides, and social value judgements documents. Versions of these guides that tailor the same information for a more specialised audience (e.g. patient/carer groups, NHS organisations), plus any amendment or addendums to these key documents, have been classed as supporting documents

#The 2001 Guidance for manufacturers and sponsors document contained detailed information on the methods of technology appraisal, much of which went on to inform the development of the first formal methods guide in 2004. As such, it has been classed here as the first edition of the methods guide

## Appendix 3: Documents coded as part of the in-depth review of TA388 (sacubitril valsartan), TA282/504 (pirfenidone) and TA297 (ocriplasmin)

**Table:**

Appraisal	Documents coded
TA388 (sacubitril valsartan)	Response to comments on draft scope Final scope Equality impact statement Committee papers, 1st meeting (pre-meeting briefing; company submission; clarification letters; addendum to company submission; consultee submissions; expert statements; evidence review group (ERG) report; ERG factual accuracy check; ERG erratum; ERG addendum) Appraisal consultation document (ACD) Committee papers, 2nd meeting (NICE’s response to comments on the ACD; new evidence submitted by company; ERG critique of new evidence) Final appraisal determination Equality impact statement (guidance development)
TA282 (pirfenidone)	Committee papers (pre-meeting briefing; manufacturer’s submission; update to the manufacturer’s submission; expert submissions; evidence review group report) Appraisal consultation document Final appraisal determination
TA504 (pirfenidone)	Draft scope Response to comments on draft scope Final scope Committee papers, 1st meeting (pre-meeting briefing; manufacturer’s submission; consultee submissions; expert statements; evidence review group (ERG) report) Appraisal consultation document (ACD) Committee papers, 2nd meeting (NICE’s response to comments on the ACD; new evidence submitted by company; ERG critique of new evidence) Committee slides, 2nd meeting Final appraisal determination (FAD) I Appeal letter Scrutiny letter Response to scrutiny letter Final scrutiny letter Appeal decision Committee papers, 3rd meeting (FAD II, appeal decision, ERG addendum) Committee slides, 3rd meeting Final appraisal determination (FAD) II Appeal letter (British Thoracic Society) Appeal letter (Roche) Scrutiny letter (British Thoracic Society) Scrutiny letter (Roche) Appeal decision Equality impact statement (guidance)
TA297 (ocriplasmin)	Final scope Equality impact assessment Evaluation report (pre-meeting briefing; manufacturer’s submission; consultee submissions; clinical expert submissions; patient expert submissions) Appraisal consultation document (ACD) NICE’s response to comments on the ACD Final appraisal determination

## Abbreviations

AfR: Accountability for reasonableness
CMS: US Centers for Medicare and Medicaid Services
FAD: Final appraisal determination
HTA: Health technology assessment
ICER: Incremental cost-effectiveness ratio
MHRA: Medicines and Healthcare Products Regulatory Agency
NHS: National Health Service
NICE: UK National Institute for Health and Care Excellence
PIM: Promising Innovative Medicine
QALY: Quality-adjusted life year
UK: United Kingdom
US: United States
